# Arabidopsis, tobacco, nightshade and elm take insect eggs as herbivore alarm and show similar transcriptomic alarm responses

**DOI:** 10.1038/s41598-020-72955-y

**Published:** 2020-10-01

**Authors:** Tobias Lortzing, Reinhard Kunze, Anke Steppuhn, Monika Hilker, Vivien Lortzing

**Affiliations:** 1 Molecular Ecology, Dahlem Centre of Plant Sciences, Institute of Biology, Freie Universität Berlin, Berlin, Germany; 2 Applied Genetics, Dahlem Centre of Plant Sciences, Institute of Biology, Freie Universität Berlin, Berlin, Germany; 3 Molecular Botany, Institute of Biology, University of Hohenheim, Stuttgart, Germany; 4 Applied Zoology/Animal Ecology, Dahlem Centre of Plant Sciences, Institute of Biology, Freie Universität Berlin, Berlin, Germany

**Keywords:** Molecular ecology, Gene ontology, Gene expression analysis, Transcriptomics, Plant ecology, Plant hormones, Plant immunity, Plant molecular biology, Plant stress responses, Secondary metabolism

## Abstract

Plants respond to insect eggs with transcriptional changes, resulting in enhanced defence against hatching larvae. However, it is unknown whether phylogenetically distant plant species show conserved transcriptomic responses to insect eggs and subsequent larval feeding. We used Generally Applicable Gene set Enrichment (GAGE) on gene ontology terms to answer this question and analysed transcriptome data from *Arabidopsis thaliana*, wild tobacco (*Nicotiana attenuata*), bittersweet nightshade (*Solanum dulcamara*) and elm trees (*Ulmus minor*) infested by different insect species. The different plant–insect species combinations showed considerable overlap in their transcriptomic responses to both eggs and larval feeding. Within these conformable responses across the plant–insect combinations, the responses to eggs and feeding were largely analogous, and about one-fifth of these analogous responses were further enhanced when egg deposition preceded larval feeding. This conserved transcriptomic response to eggs and larval feeding comprised gene sets related to several phytohormones and to the phenylpropanoid biosynthesis pathway, of which specific branches were activated in different plant–insect combinations. Since insect eggs and larval feeding activate conserved sets of biological processes in different plant species, we conclude that plants with different lifestyles share common transcriptomic alarm responses to insect eggs, which likely enhance their defence against hatching larvae.

## Introduction

Plants can boost (prime) their defences against insect herbivory when they perceive cues that indicate a risk of herbivore attack prior to the feeding damage. Among these cues are feeding- or insect egg-induced plant volatiles from neighbouring plants^1–4^, insect sex pheromones^5,6^ or insect egg deposition^7^. Plants respond to priming cues with transcriptional, phytohormonal and metabolic changes^7–9^. However, it is unknown whether plant alarm responses to herbivory-indicating cues exhibit a conformable pattern across different plant and insect species.

Insect egg deposition on plants may serve as a reliable cue for subsequent larval feeding damage. Plant responses to insect egg deposition may result in defences being mounted against the eggs^10–15^ or in enhanced defence against hatching larvae^7^. The egg-enhanced anti-herbivore defence was studied for herbaceous plant species (different Brassicaceae, *Nicotiana attenuata, Vicia faba*), the climbing bittersweet nightshade (*Solanum dulcamara*) and angio- and gymnosperm trees (*Ulmus minor* and *Pinus sylvestris*; see Table 1 for references). A plants’ response to insect eggs on its leaves alters the feeding-induced activation of phytohormone signalling pathways. While egg depositions of the butterfly *Pieris brassicae* and treatment of leaves with egg extracts lead to stronger salicylic acid (SA)-related responses in feeding-damaged brassicaceous plant species^16,17^, eggs of the moth *Helicoverpa zea* mediate a stronger jasmonic acid (JA)-related defence response in tomato plants (*S. lycopersicum*) against feeding larvae^18^. However, egg depositions by moths on *S. dulcamara* and *N. attenuata* did not affect the feeding-induced changes of JA and SA levels one day after the onset of feeding by conspecific larvae^19–22^. Prior oviposition on *N. attenuata* did, however, result in a stronger transcriptional induction of the JA-responsive transcription factor *MYB8* after feeding by larvae of the moths *Spodoptera exigua* or *Manduca sexta*^19,20^.

**Table 1:** Studies of egg-enhanced plant defence responses against chewing herbivores with special emphasis on metabolic, phytohormonal and transcriptional changes.

Plant species	Insect species	Ecological effect	Metabolic changes	Phytohormonal and transcriptional changes	References
**Brassicaceae**					
*Arabidopsis thaliana*	*Pieris brassicae*	Reduced larval weight and higher mortality	Increased levels of flavonoids Reduced levels of glucosinolates	SA: Increased SA levels Stronger expression of SA-related genes (e.g. *PR5*, *PR2*)	^17,67^
*Brassica nigra*	*Pieris brassicae*	Reduced larval weight and higher mortality Prolonged development until pupation		JA: Repressed expression of JA-related genes (e.g. *VPS2, MYC2*) SA: Increased SA levels Enhanced expression of SA-related genes (e.g. *PR2*)	^4,16,68–71^
Other Brassicaceae^a^	*Pieris brassicae*	Reduced larval weight and higher mortality Prolonged development until pupation			
**Fabaceae**					
*Vicia faba*	*Halyomorpha halys*	Reduced nymph weight			^15^
**Solanaceae**					
*Nicotiana attenuata*	*Manduca sexta*	Reduced antimicrobial activity in the hemolymph	Increased levels of caffeoyl putrescine Increased TPI activity	JA: Increased *MYB8* expression	^19,20^
	*Spodoptera exigua*	Reduced larval weight and higher mortality	Increased levels of caffeoyl putrescine	JA: Increased *MYB8* expression	
*Solanum lycopersicum*	*Helicoverpa zea*			JA: Increased JA levels Enhanced expression of *PIN2*	^18^
*Solanum dulcamara*	*Spodoptera exigua*	Reduced larval weight and higher mortality	Stronger expression of genes involved in the phenylpropanoid pathways, e.g. anthocyanins	JA, SA and ABA levels were not affected by prior egg deposition	^22^
**Ulmaceae**					
*Ulmus minor*	*Xanthogaleruca luteola*	Higher larval mortality	Increased uptake of robinin (flavonoid) by the larvae Faster/earlier expression of defence-related genes		^24,55^
**Pinaceae**					
*Pinus* *sylvestris*	*Diprion pini*	Reduced larval weight and higher mortality Reduced female fecundity			^72^

^a^*Brassica oleraceae, Sinapis arvensis* and *Moricandia moricandioide.*

Until now, it has been unclear whether plant alarm responses elicited by insect eggs lead to conformable transcriptional changes across different plant and insect species. Therefore, we asked whether different plant species show conformable transcriptional responses to insect eggs as a general alarm cue indicating herbivory. We further investigated whether the egg-mediated alarm responses alter the transcriptional responses of the plant species to feeding larvae in a conformable manner. This comparison of transcriptomic plant responses to insect eggs and larval feeding across different plant and insect species has the potential to reveal conserved response patterns fundamental to the plants’ anti-herbivore defences.

**Figure 1:**
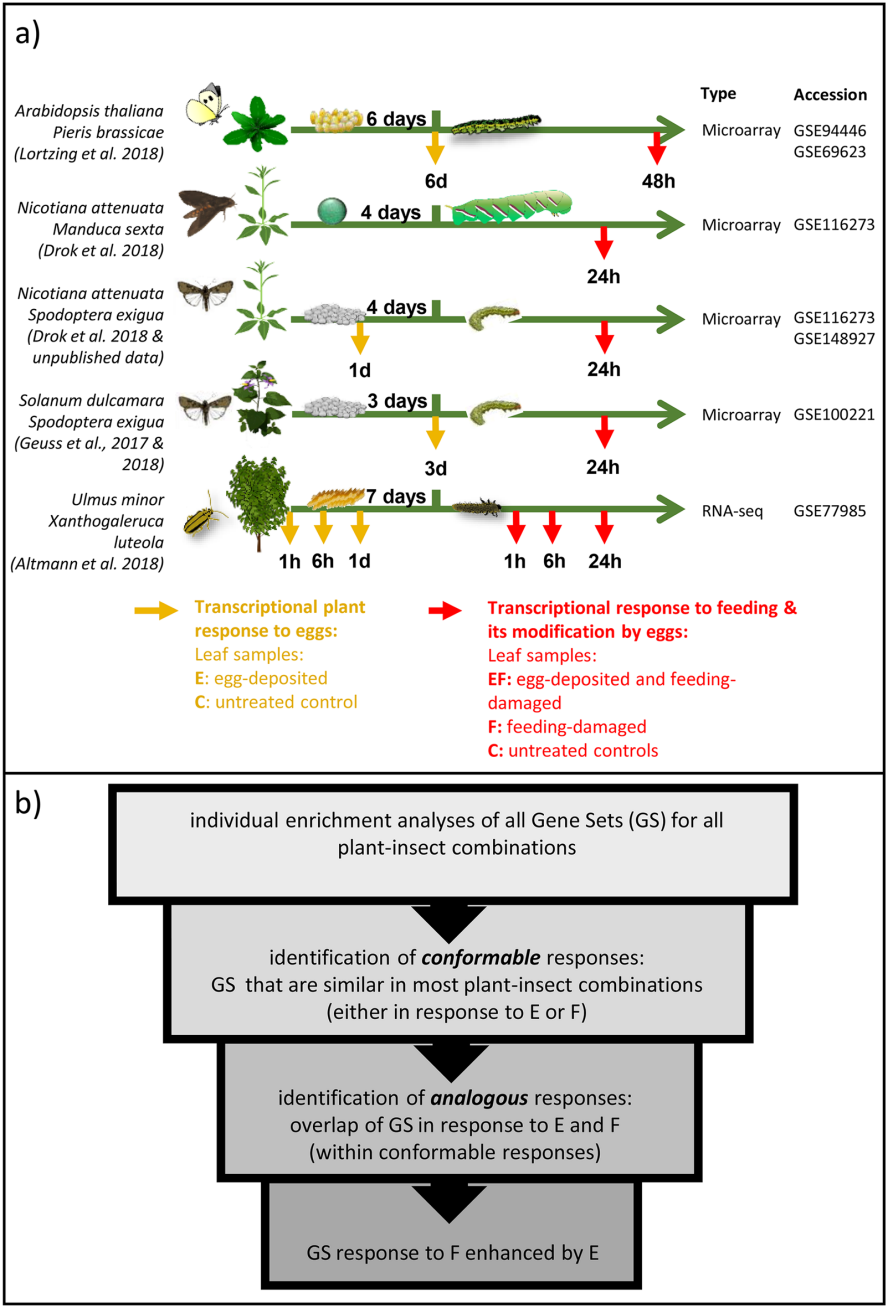
Overview of the five plant–insect combinations investigated, of the experimental setups of the respective transcriptome studies and of the GAGE workflow. (**a**) Experimental setups of transcriptome analysis studies on plants exposed to insect eggs, larval feeding, or eggs and feeding. Yellow arrows show harvest time points for investigating leaf material for the effects of egg deposition on the plant transcriptome; leaf material was harvested from insect egg-deposited (E) and untreated control plants (C). Red arrows represent the harvest time points for investigating leaf material for the effects of larval feeding; leaf material was harvested from egg-deposited and larval feeding-damaged (EF), egg-free, feeding-damaged (F) and untreated control plants (C). All data are available on the NCBI Gene Expression Omnibus repository according to the accession numbers. For further details on experimental setups see “Materials and methods”. (**b**) Workflow applied to identify gene sets (GS) that show an altered expression response to either insect eggs (E) or to larval feeding (F), which are conformable across the plant–insect combinations. From these conformable GS, we identified those that react analogously to E and F or that show an enhanced response to F when plants previously experienced E.

We used standardised Generally Applicable Gene set Enrichment (GAGE) analyses^23^ on gene ontology (GO) terms to investigate which plant biological processes (BPs) are mainly affected in response to (i) insect eggs, (ii) feeding by neonate larvae, and (iii) insect eggs followed by larval feeding (Fig. 1a). For the analysis we used transcriptomic data from published experiments with *A. thaliana* infested by *P. brassicae*^17^, *N. attenuata* infested by *M. sexta* or by *S. exigua*^21^, *S. dulcamara* infested by *S. exigua*^12,22^ and *U. minor* infested by the leaf beetle *Xanthogaleruca luteola*^24^. Thus, our analysis is based on transcriptomic data obtained by studies of different plant–insect combinations, including plant species with very different life strategies, as well as lepidopteran and coleopteran insect species. While the lepidopteran species do not damage the leaf when laying their eggs, the coleopteran species (*X. luteola*) slightly damages the host leaf by removing the epidermal cell layer at the site where eggs are laid. As the experimental conditions between the studies differ substantially, and as the GAGE algorithm can potentially generate relatively high false-positive rates, this analysis is not suitable to draw conclusions about single responses, which are specific to an individual plant–insect combination. However, transcriptional patterns that emerge as conserved between the different systems despite experimental differences are likely at the core of plant responses to insect eggs and feeding and thus primary targets for detailed analyses. We aim to figure out whether different plant species show a core set of transcriptomic herbivore alarm responses, which are phylogenetically conserved across the investigated plant species. Different genome sizes, different sizes of gene families, and inaccurate annotations on the level of individual genes in most species hinder comparisons of transcriptomic data from different plant species at the level of individual genes. Therefore, we compared the results of these studies at the functional level of biological processes to elucidate conformable plant response patterns to insect eggs, larval feeding and insect eggs with subsequent larval feeding across these different plant and insect species.

Classical GO term enrichment, used in the earlier original publications about the responses of the afore mentioned plant species, compares gene frequencies in GO terms between a list of significantly regulated genes and a list of all genes included in the expression analysis. In contrast, the GAGE approach includes the quantitative change in expression of all genes in a given gene set (GS) and calculates an enrichment score based on the overall change in expression of genes within this GS^23^. Using GO term annotation to generate GS for GAGE utilises more data than classical GO term enrichment and allows for a standardised statistical analysis of transcriptional changes in functional groups with data from different species and from different sources.

We compared the transcriptomes of the five aforementioned plant–insect combinations (Fig. 1) with respect to the following questions: (i) Do insect eggs and feeding larvae, respectively, elicit transcriptional effects on plant biological processes that are conformable across the different plant–insect combinations? (ii) What is the overlap of transcriptional responses to egg deposition and to feeding larvae within these conformable responses? (iii) Which of the conformable responses show additive, or even synergistic, effects when egg deposition precedes insect feeding?

Figure 1b illustrates the general workflow of the analysis. We first identified for each treatment those GS that responded similarly across most plant–insect combinations. As such, we classified all GS with a FDR-adjusted *p*-value < 0.05 in three out of four plant–insect combinations for the egg deposition treatment, and in four out of five combinations for insect feeding, either with or without prior egg deposition. We will refer to response patterns that are similar across the different plant–insect systems as *conformable* responses.

Subsequently, we searched for the overlap of GS regulation between the different treatments within these conformable responses. We will refer to this overlap between treatments as *analogous* responses. The earlier original publications describing transcriptional analyses of the five plant–insect combinations showed that feeding-induced plant responses enhanced by prior egg deposition are linked with transcriptional regulation of phytohormonal signalling pathways and of the phenylpropanoid pathway. Our analyses largely corroborate these findings. Therefore, we proceeded to analyse these biological processes with a detailed, targeted approach.

## Results and discussion

### Biological processes involved in plant responses to insect eggs

We first explored whether the investigated plant species show conformable transcriptional reprogramming in response to insect eggs.

**Figure 2:**
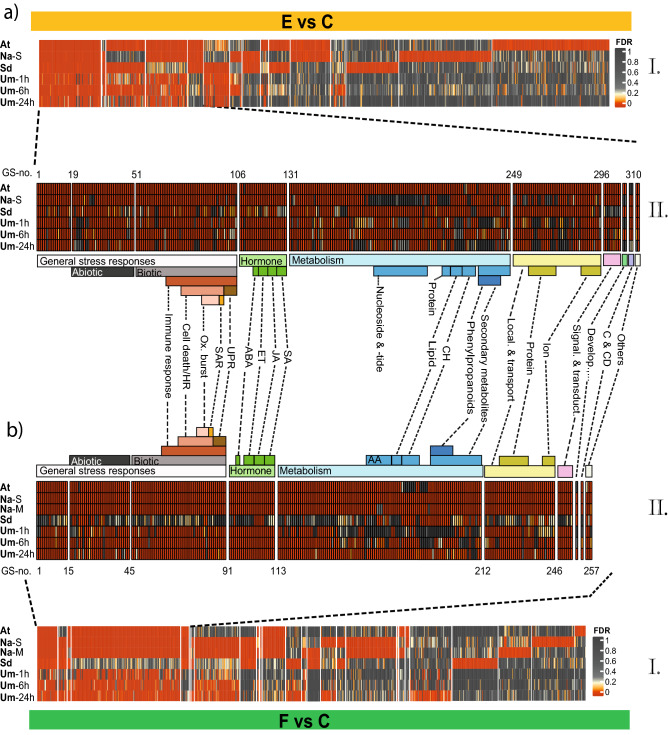
Comparison of GAGE analyses of the transcriptional up-regulation in four different plant species in response to (**a**) insect eggs (E vs. C) and (**b**) larval feeding (F vs. C). The species are given below^†^. The heatmaps depict false discovery rate-adjusted *p*-values (FDR) according to the colour key of up-regulated gene sets (GS): I. GS significantly enriched in at least one plant species, II. Heatmap sections of the conformably enriched GS in at least three out of four (**a**) and four out of five (**b**) plant–insect interactions. GS in II were re-ordered according to the biological function. For detailed descriptions of the GS see Supplementary Table S2 (E vs. C up and F vs C up). AA: amino acid; ABA: abscisic acid; C & CD: cytokinesis & cell differentiation; CH: carbohydrates; Develop., …: development, morphogenesis & reproduction; ET: ethylene; JA: jasmonic acid; Local.: localisation; Ox. burst: oxidative burst; SA: salicylic acid; SAR: systemic acquired resistance; Signal. & transduct.: signalling and transduction; UPR: unfolded protein response. ^†^At: *Arabidopsis thaliana-Pieris brassicae*; Na-M: *Nicotiana attenuata-Manduca sexta*; Na-S: *N. attenuata-Spodoptera exigua*; Sd: *Solanum dulcamara-S. exigua*; Um-1 h/-6 h/-24 h: *Ulmus minor-Xanthogaleruca luteola* after 1/6/24 h of egg deposition or larval feeding. For detailed experimental setup description see Fig. 1.

Taking into account all GS included in the analysis for all plant–insect combinations tested and significantly regulated in at least one of them, we found down-regulation of 649 and up-regulation of 969 GS in response to egg deposition (Supplementary Table S1). Of these, we identified an overlap of 52 down-regulated and 310 up-regulated GS with conformable regulation across the different plant–insect combinations (Fig. 2a, Supplementary Table S2). This indicates that plant species with very different lifestyles share up to 32% of transcriptional regulation in biological processes after egg deposition from different insect species.

The 52 down-regulated GS represent 8% of conformable regulation across the plant–insect combinations and were mostly related to regulation of gene expression and some developmental, morphological and cell cycle processes (Supplementary Table S2).

Of the 310 conformably up-regulated GS, 34% belong to generic stress responses, roughly equally distributed between abiotic and biotic stress responses (Fig. 2a, Supplementary Table S2). The majority of GS associated with “biotic stress” were related to plant immune responses, which comprise hypersensitive response (HR)-like responses and cell death, accumulation of reactive oxygen species (ROS), systemic acquired resistance (SAR), endoplasmic reticulum stress and unfolded protein responses (UPR). Insect egg depositions can cause obvious phenotypic leaf tissue modifications, such as necrosis/chlorosis and neoplasm formation at the site of egg deposition. These egg-induced leaf modifications occur in several plant species, including *A. thaliana*, *Brassica nigra*, *S. dulcamara* and *P. sylvestris,* thus resembling a HR-like symptom, which is linked to the accumulation of ROS^11–13,25–27^. This egg-induced change in leaf traits might result in egg desiccation or detachment of eggs from leaves. Some plant species may rely on ROS signalling to initiate formation of chlorotic or necrotic leaf tissue at the site of egg deposition^10^; others use extensive ROS accumulation to directly kill the eggs^12^. An oxidative burst is an essential signalling component for the formation of necrotic lesions, which are typical for HR-like responses. To our surprise, plant species like tobacco and elm, which do not show obvious HR-like symptoms in response to eggs, display transcriptional activation of innate immune responses similar to that of *A. thaliana* and *S. dulcamara* (Fig. 2a).

Further conformably up-regulated GS after egg deposition included, among others, GS involved in small and macromolecule metabolism, metabolism of organic acids, amines, cyclic carbohydrates and phenylpropanoids, and GS related to phytohormones (Fig. 2a).

**Figure 3:**
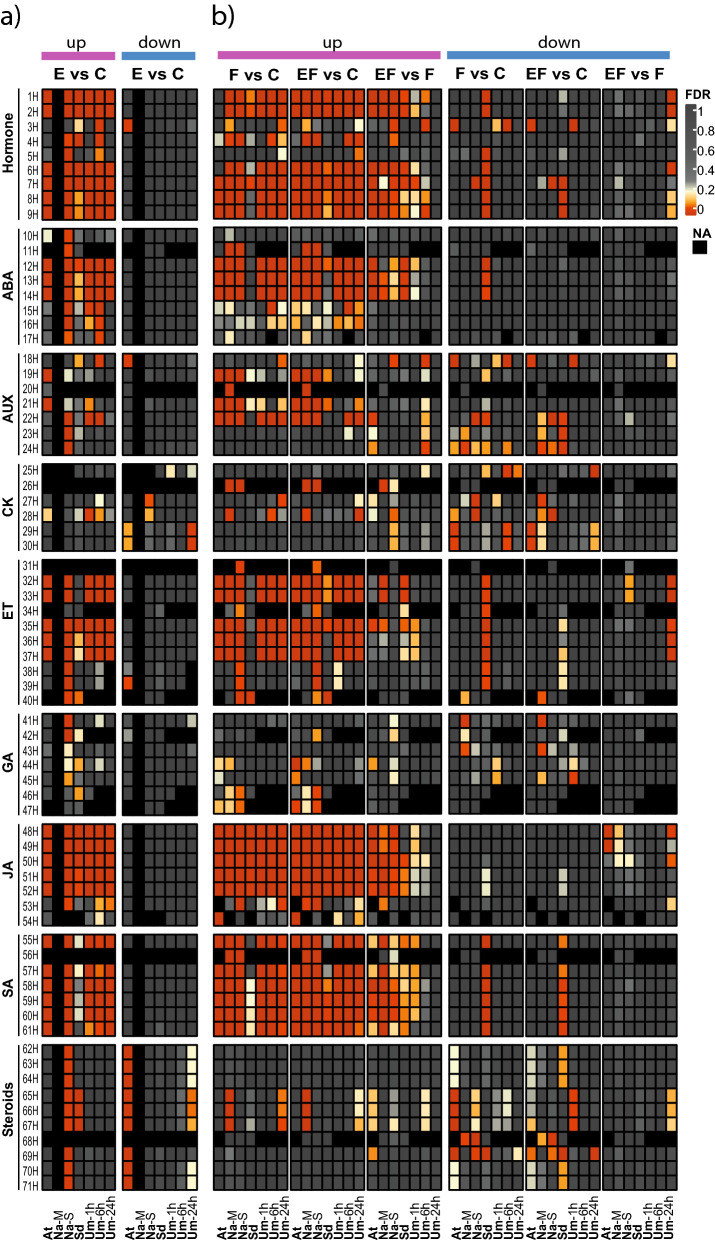
Comparison of GAGE analyses of phytohormones from five different plant–insect combinations^†^ depicting plant responses to (**a**) insect eggs (E vs. C) and (**b**) larval feeding (F vs. C), eggs with subsequent feeding (EF vs. C) and the alterations in plant responses to feeding by prior egg deposition (EF vs. F). The heatmap depicts false discovery rate-adjusted *p*-values (FDR) according to the colour key for up- or down-regulated gene sets (GS). Black boxes indicate GS which could not be assigned to the plant species or for which enrichment scores were not calculated due to a lack of data (E vs. C; Na-M). For a detailed description of the phytohormone-related GS 1H-71H (H) see Supplementary Table S3. ABA: abscisic acid; AUX: auxin; CK: cytokinin; ET: ethylene; GA: gibberellic acid; JA: jasmonic acid; SA: salicylic acid. ^†^At: *Arabidopsis thaliana-Pieris brassicae*; Na-M: *Nicotiana attenuata-Manduca sexta* Na-S: *N. attenuata-Spodoptera exigua*; Sd: *Solanum dulcamara-S. exigua*; Um-1 h/-6 h/-24 h: *Ulmus minor-Xanthogaleruca luteola* after 1/6/24 h of egg deposition and larval feeding, NA: not annotated. For a detailed experimental setup description see Fig. 1.

In response to pathogens, ROS synergistically amplify the SA signal to induce HR-like symptoms and the expression of *PATHOGENESIS-RELATED (PR)* defence genes such as *PR1*^e.g.^^28–30^. *PR* genes are also more strongly expressed in response to eggs in several plant species^12,15,31,32^. We found strong conformable up-regulation of SA-related GS in response to eggs (Fig. 3a, Hormones (H); for abbreviations see Supplementary Table S3). This effect is quite weak only in *S. dulcamara*, although this plant species accumulates SA in response to insect eggs, as has been shown in phytohormone measurements by Geuss et al.^12^. Hence, the ROS- and SA-mediated induction of immune responses and *PR* gene expression in response to eggs is conserved amongst different plant species and both might contribute to direct plant defence against insect eggs. Interestingly, JA, abscisic acid (ABA) and ethylene (ET) signalling are also part of the conformable response to insect eggs (Fig. 2a and Fig. 3a).

Taken together, the different plant–insect combinations showed a considerable overlap in their transcriptomic responses to insect eggs, including up-regulation of GS related to generic stress responses and down-regulation of GS related to development and cell cycle processes. This would suggest a conserved plant response to insect egg depositions, regardless of whether the egg deposition is associated with leaf damage (as is the case for *U. minor*).

### Biological processes involved in plant responses to insect feeding and their similarities to plant responses to insect eggs

Using the same methodology as for the E vs. C comparison, we searched for conformable plant transcriptional responses to feeding herbivores (F vs. C) across the plant–insect combinations we investigated. Then, we compared the conformable responses to feeding and to insect eggs with each other to identify a subset of GS that responds analogously in both treatments.

Larval feeding led to down-regulation of 972, and to up-regulation of 911 GS in at least one of the plant–insect combinations (Supplementary Table S1).

**Figure 4:**
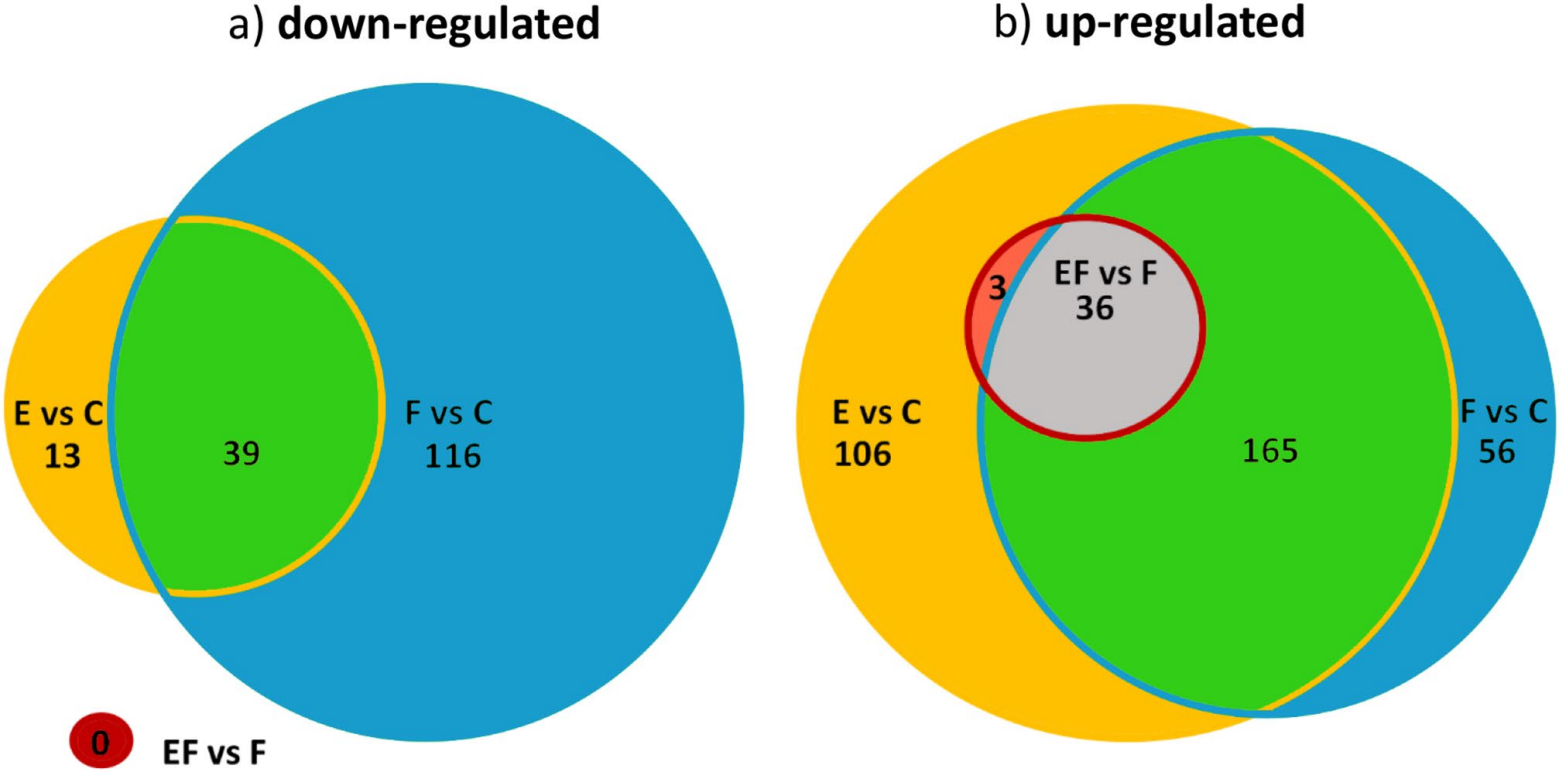
Venn diagrams with the number of gene sets (GS) that showed conformable (**a**) down-regulation and (**b**) up-regulation across the different plant–insect combinations when comparing the plant response to eggs (E vs. C, Fig. 2a II), to feeding (F vs. C, Fig. 2b II) and to eggs followed by feeding (EF vs. F, Fig. 5b). For detailed descriptions of uniquely or commonly enriched GS see Supplementary Table S4.

Of the down-regulated GS, 16% were conformably down-regulated (Supplementary Table S2). Similar to the response to eggs, the feeding-responsive down-regulated GS included especially those associated with regulation of gene expression by epigenetic and post-transcriptional modifications, developmental and morphological processes and cell cycle processes (Fig. 4a, green intersection, Supplementary Tables S2 and S4).

A considerable fraction of GS (28%) was conformably up-regulated in response to feeding (Fig. 2b, Supplementary Table S2). The vast majority (78%) of these GS overlapped with the conformable response to eggs (Fig. 4b, green intersection). The GS in this analogous response to eggs and to feeding included most stress- and plant immune response-related GS like ROS production, phytohormonal regulation and large parts of the metabolism-related GS, e.g. biosynthesis of aromatic compounds and phenylpropanoid metabolism (Supplementary Table S4), but lacked the GS related to nucleoside/-tide metabolism, which responded only to egg deposition.

As expected^33^, the conformable plant response to feeding includes many JA-related processes, accompanied by ABA and ET signalling. However, we also found a surprisingly consistent enrichment of GS related to immune responses, SA and ROS signalling in feeding-induced leaves (Figs. 2b and 3b).

In all of the plant species investigated here, JA-related responses dominated the plant response to feeding^e.g.^^12,34^. However, some studies found SA levels to be slightly enhanced after herbivory by *P. brassicae* in *A. thaliana*^17^ and by *M. sexta* and *S. exigua* in *N. attenuata*^35,36^, but not in *S. dulcamara*^37^. Elevated SA levels frequently antagonise JA-mediated plant defences against herbivory^38,39^; they are therefore usually considered to be beneficial for chewing herbivores^e.g.^^40^. However, activation of SA signalling is not always advantageous for the herbivore^16,17,41^. JA and SA are embedded in a complex phytohormonal signalling network which determines, as a whole, the metabolic outcome affecting biotic stressors like insects^33^. Subtle changes in SA levels may therefore fine-tune a JA-dominated response within this phytohormonal network and vice versa^42^.

Overall, the conformable feeding-induced transcriptional response observed in the different plant–insect combinations was remarkably similar to the conformable response to insect egg deposition. Developmental, morphogenesis and growth processes were down-regulated in response to eggs and feeding, indicating that metabolic resources might be shifted towards defence and stress reaction (Fig. 4a, Supplementary Table S4). The up-regulation of immune-related stress responses, phytohormonal regulation and secondary metabolism-related GS were almost identical in the conformable responses to egg deposition and to feeding (Fig. 2). The particularly large overlap in ROS-related stress responses and the involvement of multiple phytohormonal signalling pathways might indicate a more fundamental role of ROS signalling in plant responses to insect eggs beyond the formation of defensive HR-like symptoms. ROS are not only important as a second messenger during establishment of HR, but are closely connected with the hormonal signalling network and metabolic reprogramming after herbivore attack^43,44^.

### Modification of plant transcriptional responses to larval feeding by prior egg deposition

A comparison of the transcriptomes of feeding-damaged plants with and without prior egg deposition (EF vs. F) revealed 84 down-regulated and 630 up-regulated GS in at least one of the plant–insect combinations (Supplementary Table S1).

**Figure 5:**
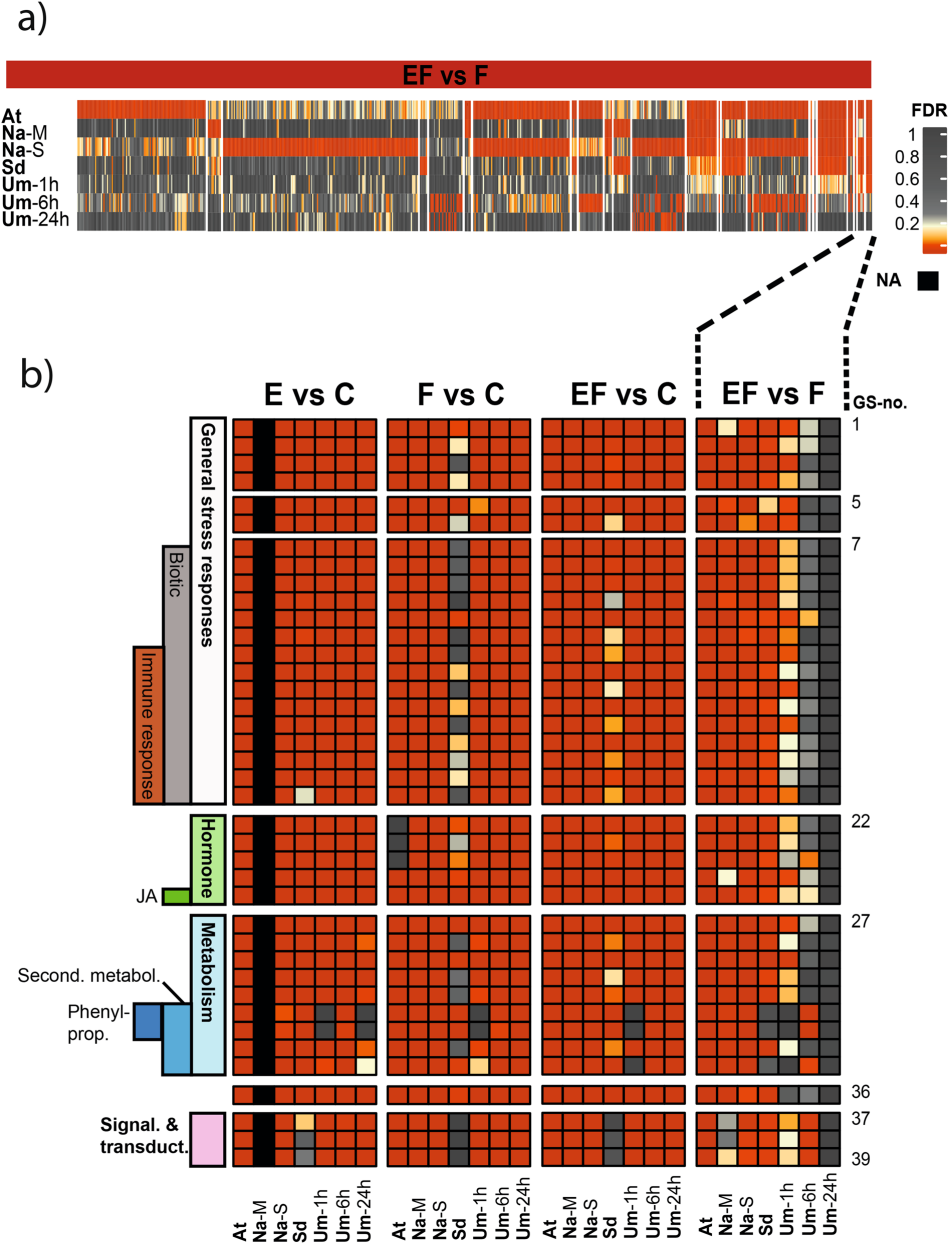
Comparison of GAGE analyses of the transcriptional up-regulation in four different plant species comparing (**a**) the response to larval feeding with and without prior egg deposition (EF vs. F; species are given below^†^). The heatmap depicts false discovery rate-adjusted *p*-values (FDR) according to the colour key of all up-regulated gene sets (GS) significantly enriched in at least one plant species; (**b**) Heatmap-section of a) with conformably enriched GS in at least four out of five plant–insect combinations (EF vs. F) and FDR values of the same GS in comparisons between untreated controls and egg-deposited (E vs. C), feeding damaged (F vs. C) or egg deposited and feeding damaged (EF vs. C) plants. GS in b) were re-ordered according to their biological function. For detailed descriptions of the GS see Supplementary Table S2 (EF vs. F up). JA: jasmonic acid; Phenylprop.: phenylpropanoids; Second. metabol.: secondary metabolites; Signal. & transduct.: signalling and transduction. ^†^At: *Arabidopsis thaliana-Pieris brassicae*; Na-M: *Nicotiana attenuata-Manduca sexta* Na-S: *N. attenuata-Spodoptera exigua*; Sd: *Solanum dulcamara-S. exigua*; Um-1 h/-6 h/-24 h: *Ulmus minor-Xanthogaleruca luteola* after 1/6/24 h of egg deposition and larval feeding, respectively, NA: not available. For detailed experimental setup description, see Fig. 1.

We did not detect any conformably down-regulated GS (Supplementary Table S2), whereas 39 GS were conformably up-regulated across the plant–insect combinations (Fig. 5a, Supplementary Table S2). Almost all (36) of the latter GS were also found in the analogous responses to feeding and eggs (Fig. 4b, grey intersection, Supplementary Table S4). They account for a core set of 18% of the GS analogously regulated by eggs and by feeding in most of the plant species we tested. These GS indicate additive or synergistic effects when egg deposition precedes larval feeding. It includes mostly biotic stress and immune responses with regulation of cell death, but also hormonal responses, particularly the response to JA and phenylpropanoid biosynthesis (Fig. 5b, Supplementary Table S2).

In summary, a considerable percentage of the activated GS involved in the analogous egg and feeding responses is further enhanced when plants experience both stimuli in succession. This suggests a conserved herbivore alarm response that is initiated by insect egg deposition and affects the transcriptional response induced by feeding in an additive or synergistic manner.

### The plant’s transcriptional response to eggs, larval feeding and to the combination of eggs followed by larval feeding involves several phytohormone pathways

Our analysis, and the earlier original publications^12,17,21,22,24^ to which our analysis refers, found prominent regulation of GS related to phytohormone signalling. Therefore, we compared the enrichment of all GS associated with phytohormone signalling and metabolism that were up- or down-regulated in at least one of the species combinations (Fig. 3, Supplementary Table S3).

In response to eggs (Fig. 3a, E vs. C), many GS related to generic hormone responses were up-regulated in most plant–insect species combinations. GS related to JA, SA, ABA and ET follow this pattern. GS related to other phytohormones showed a more differentiated response pattern. A few auxin (AUX)-related GS were up-regulated, but their number differed between the plant–insect combinations. Gibberellic acid (GA)- and steroid-related GS were up-regulated in *N. attenuata,* but the latter were down-regulated in *A. thaliana*. *Solanum dulcamara*’s response to eggs involved a less clear enrichment of the ET- and SA-related GS than the responses to eggs by the other plant species.

Elevated transcription of SA-related GS in response to insect eggs has frequently been described but the conserved induction of JA-, ET-, and ABA-related GS is surprising. Enhanced activation of JA signalling is plausible for *U. minor* because the leaves in this dataset were mechanically wounded to mimic the leaf damage inflicted by the beetles during egg deposition^24^. The wound stimulus alone might have elicited the induction of JA-mediated pathways in the egg treatment^45,46^. Egg deposition in *U. minor* also elicits the emission of terpene volatiles, which is frequently linked to JA-dominated signalling events^47^. However, all of the other plant species we analysed also showed activation of JA-related GS in response to lepidopteran egg deposition, which does not damage any plant tissue (Figs. 2a and 3a). Some studies indicate that egg deposition without tissue damage might indeed elicit JA-related responses in plants. *Solanum lycopersicum* enhances the expression of a proteinase inhibitor (PI) gene in response to *H. zea* eggs, which correlates with increased JA levels^18^, and lepidopteran egg deposition on *S. dulcamara* results in JA-dependent enhanced leaf PI activity^12^, which is also linked to ABA and ET signalling^37,48^.

The distinct egg-induced changes in the expression of GS related to phytohormones suggest that plant responses to egg deposition do not only rely on SA- and ROS-related responses. Egg deposition rather causes a complex reorganisation of the dynamic phytohormonal signalling network, which is remarkably similar across the different plant–insect combinations.

The plant responses to larval feeding (Fig. 3b, F vs. C, up) involved similar phytohormonal GS as the responses to the eggs. They included strong up-regulation of JA-, SA-, ABA- and ET-related GS in most plant–insect combinations. The only exception was *S. dulcamara*, which showed strong up-regulation of JA-related GS, but clear down-regulation of ABA-, SA- and ET-related GS (Fig. 3b, F vs. C, down), which could indicate a weaker inducible response to larval feeding in this species, which maintains a quite effective constitutive defence due to its high content of steroidal alkaloids. Alternatively it might be a side effect of weaker feeding damage in the experimental setup used (see “Methods” section, Additional evaluation of *Solanum dulcamara* transcriptome data). In general, more AUX-related GS were up-regulated in response to feeding than in response to eggs.

Insect egg deposition enhanced the hormonal plant response to larval feeding (Fig. 3b, EF vs. F, up). The feeding-induced up-regulation of JA- and SA-related GS was further enhanced by prior egg deposition in all plant–insect combinations, although egg-treated elms showed this enrichment only at the onset of larval feeding (after 1 h), but not later on. ABA- and, to a lesser degree, ET-related GS were also commonly up-regulated when egg deposition preceded larval feeding. In *U. minor*, egg deposition also caused down-regulation of a fraction of genes in ET-related GS and further enrichment of down-regulated ET-related GS after 24 h of feeding (Fig. 3b, EF vs. F, down). Although in *S. dulcamara* phytohormonal regulation was only moderately affected by egg deposition and feeding alone*,* the combination of the two treatments led, as in the other plant–insect combinations, to increased expression of genes in ABA-, ET-, JA- and SA-related GS. In *N. attenuata*, feeding-induced expression of ET-related GS was only enriched after *M. sexta* egg deposition.

Taken together, JA-, ABA- and SA-related GS were more strongly enriched in all plant–insect combinations when egg deposition preceded larval feeding (Fig. 3). ET-related GS were strongly affected by both stimuli alone, but showed only faint additive or synergistic responses when eggs preceded larval feeding. Previous studies had already suggested that either JA- or SA-mediated pathways are further intensified in response to feeding when egg deposition occurs prior to larval feeding (Table 1). Our analysis corroborates these findings but furthermore suggests an interplay of several phytohormones mediating the improved anti-herbivore defence in egg-deposited and subsequently feeding-damaged plants. Although egg deposition and larval feeding are very different stimuli, it becomes quite clear that both affect the phytohormonal network at the transcriptional level in a similar way. Egg depositions and larval feeding may therefore trigger similar changes in metabolism-related GS across the plant–insect combinations (Fig. 2), which might contribute to the enhanced anti-herbivore defence we observed following egg deposition.

### Phenylpropanoid metabolism and its involvement in the egg-mediated plant defence response to larvae

**Figure 6:**
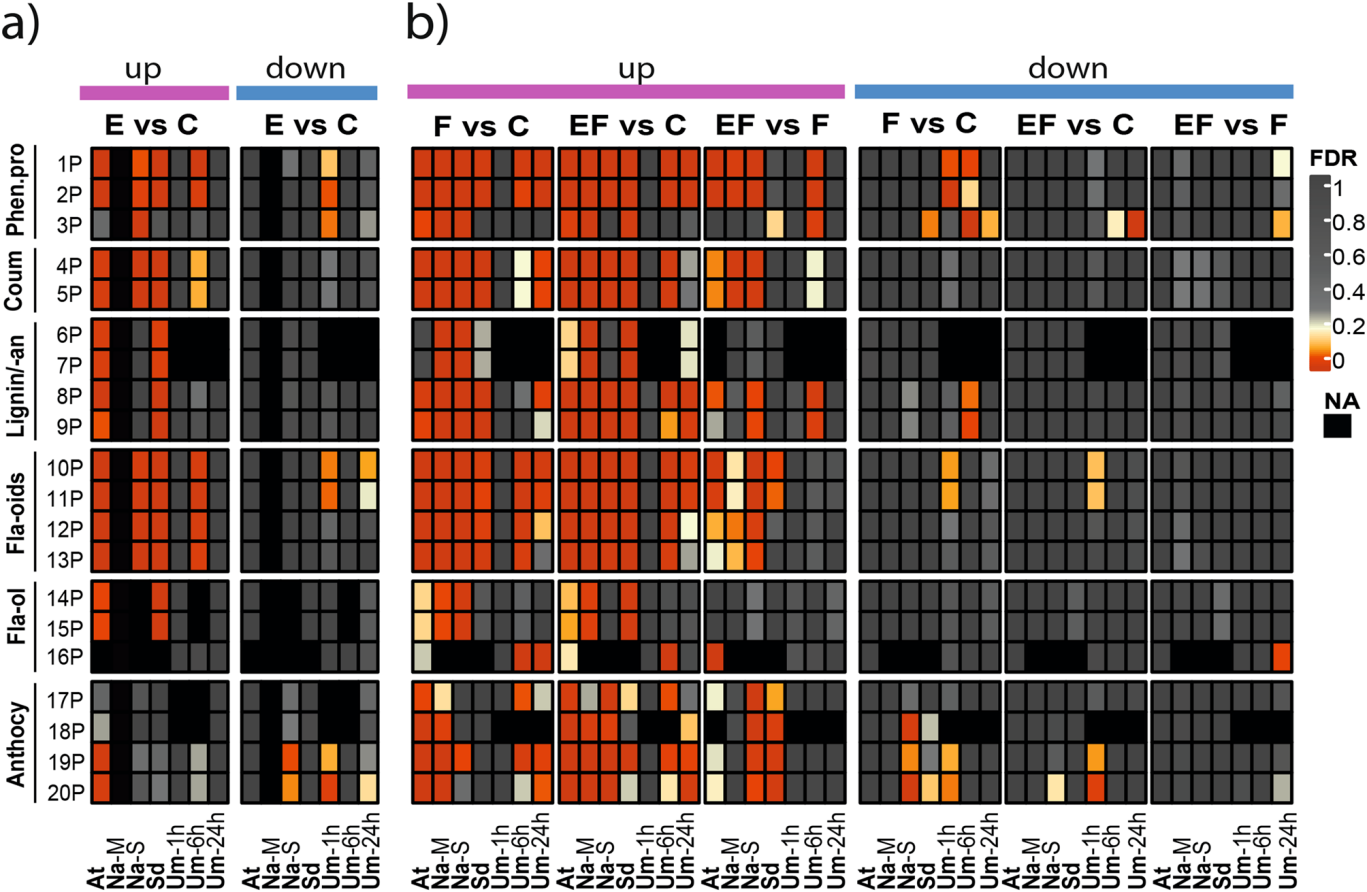
Comparison of GAGE analyses associated with the phenylpropanoid pathway between five different plant–insect combinations^†^, depicting plant responses to (**a**) insect eggs (E vs. C) and (**b**) larval feeding (F vs. C), eggs with subsequent feeding (EF vs. C) and the alterations in plant responses to feeding by prior egg deposition (EF vs. F). The heatmap depicts false discovery rate-adjusted *p*-values (FDR) according to the colour key for up- or down-regulated gene sets (GS). Black colour indicates GS which could not be assigned to the plant species or for which enrichment scores were not calculated due to a lack of data (E vs. C; Na-M). For a detailed description of the 20 phenylpropanoid pathway-associated GS (1P-20P) see Supplementary Table S3. Phen.pro: phenylpropanoids; Coum: coumarins; Fla-oids: flavonoids; Fla-ol: flavonols; Anthocy: anthocyanins. ^†^At: *Arabidopsis thaliana-Pieris brassicae*; Na-M: *Nicotiana attenuata-Manduca sexta* Na-S: *N. attenuata-Spodoptera exigua*; Sd: *Solanum dulcamara-S. exigua*; Um-1 h/-6 h/-24 h: *Ulmus minor-Xanthogaleruca luteola* after 1/6/24 h of egg deposition and larval feeding, respectively, NA: not annotated. For detailed experimental setup description, see Fig. 1.

The results of the plant–insect combinations studied here suggest that regulation of the phenylpropanoid pathway is linked to the impaired performance of herbivores on previously egg-deposited plants (Table 1). Our analysis also shows that induction of the phenylpropanoid pathway by feeding damage was enhanced by prior egg deposition. Phenylpropanoids are well known for their diverse roles in anti-herbivore defence^49–51^. The phenylpropanoid pathway is widely branched^52^, and each branch leads to end products which may impair feeding herbivores^e.g.^^53,54^. We applied GAGE to evaluate the transcriptional regulation of those pathway branches that were regulated in at least one of the plant–insect combinations. In this way we could determine whether certain branches of the phenylpropanoid pathway showed analogous regulation patterns that could explain the egg-mediated enhancement of the plant’s defence against feeding herbivores across the different plant–insect combinations (Fig. 6, Phenylpropanoids (P); for abbreviations see Supplementary Table S3).

In response to eggs (Fig. 6a, E vs. C, up), we found conformable up-regulation of generic phenylpropanoid-related GS across all of our plant–insect combinations. These GS are mainly coumarin- and flavonoid-related. *Ulmus minor* showed up-regulation of flavonoid-related GS only after 6 h of egg deposition, although some of them were down-regulated after 1 h and after 24 h. In addition, lignin-related GS were clearly up-regulated after egg deposition in *A. thaliana* and *S. dulcamara*.

In response to feeding (Fig. 6b, F vs. C), GS in all branches of the phenylpropanoid pathway were up-regulated in almost all of the plant–insect combinations tested, with *S. dulcamara* being the only exception that did not show a response in its flavonol and anthocyanin-related GS. The response of *N. attenuata* to the herbivore species studied (*M. sexta, S. exigua*) differed with respect to the regulation of anthocyanin-related GS. Feeding by *M. sexta* led to up-regulation, but the response to feeding by *S. exigua* resulted in a more diffuse response pattern with less clear up-regulation, and even some down-regulation, of those GS. The late feeding-induced up-regulation of the phenylpropanoid-related GS in *U. minor* illustrates that there is a lag between the onset of feeding and the induction of this pathway, and that it is apparent even at the transcriptomic level.

When egg deposition preceded larval feeding (Fig. 6b, EF vs. F, up), pronounced up-regulation of several of the phenylpropanoid-related GS was found in all plant–insect combinations in response to feeding. This egg-enhanced response to feeding primarily affected GS related to flavonoids and anthocyanins in *S. dulcamara* and *A. thaliana.* Previously egg-deposited *A. thaliana* and *U. minor* both showed enhanced transcription in lignin-related processes in response to feeding damage. In *N. attenuata,* we found enhanced up-regulation of coumarin- and anthocyanin-related GS after egg deposition and feeding by *S. exigua*, but not by *M. sexta.* Interestingly, *S. exigua* feeding alone (without prior egg deposition) hardly induced any anthocyanin-related responses. Insect egg deposition on *U. minor* resulted in a stronger feeding-induced up-regulation of GS linked to lignin-related processes after a 6 h feeding period.

Our analysis shows that egg-enhanced activation of phenylpropanoid-related gene expression after feeding is indeed a conserved response across the plant species investigated. Metabolite analyses in *A. thaliana* showed increased flavonol levels in egg-deposited and feeding-damaged plants, while in *N. attenuata* caffeoylputrescine, a phenylpropanoid-polyamine conjugate, was found to be responsible for the reduced performance of *S. exigua* on egg-deposited plants^17,19,20^. Larvae of the elm leaf beetle suffered higher mortality on previously egg-deposited elm, and this was accompanied by an increased uptake of a flavonoid (kaempferol 3-*O*-robinoside-7-*O*-rhamnoside)^55^.

The huge diversity in plant secondary metabolites, including phenylpropanoids, likely facilitates plant defence as it hampers the counter-adaptations of herbivores feeding on those plants^56^. It is almost certain that each of the distantly related host plants investigated here holds a different phenylpropanoid profile. Almost all branches of the phenylpropanoid pathway in all plant–insect combinations were feeding-induced, but the modification of this induction profile by eggs was specific to the plant–insect combination analysed.

It appears that the egg-mediated modification of feeding-induced gene expression in the phenylpropanoid pathway in general is a conserved response, but the specific branches of this pathway seem to be affected in a plant-, and perhaps even herbivore-, specific way. Accordingly, plants might use the egg stimulus not only to prepare against impending herbivory in general, but to fine-tune the feeding-induced phenylpropanoid defences according to the specific herbivore they are likely to encounter. This idea is further supported by the finding that *N. attenuata* exhibits altered transcriptomic responses to feeding by *S. exigua* and *M. sexta* when the plant has received the eggs of the respective other herbivore prior to feeding^21^.

## Conclusion

The different plant species show strong transcriptomic responses to insect eggs and larval feeding. At the functional-transcriptomic level, distantly related plant species with different lifestyles show conformable activation of about 30% of the biological processes in response to insect eggs and feeding, respectively. The biological processes represented in these two phylogenetically conserved conformable responses overlapped to 78%, which was independent of the plant species and the infesting insect species. Around one-fifth of the biological processes within this overlap was more strongly regulated when plants experienced insect egg deposition and larval herbivory in succession. As such, the plants investigated seem to possess a conserved transcriptional core response to herbivore attack, which includes regulatory and metabolism-related biological processes and which is initiated as soon as an herbivorous insect lays its eggs.

The considerable overlap in conserved plant responses to insect eggs and feeding, as well as the egg-enhanced regulation of a significant portion of them, supports the hypothesis that plants use insect eggs as an alarm cue to prepare themselves for defence against the hatching herbivorous larvae, which triggers the plant defences to their full extent. This egg-enhanced alarm response against the feeding larvae might be based on a metabolic shift of resources towards defence by down-regulation of developmental and cell cycle processes established during the plant response to eggs and feeding alone.

Whether the perception of eggs induces defences similar to those triggered by larval feeding, or rather accelerates, amplifies, or fine-tunes the defence response to a feeding herbivorous insect, needs to be addressed in future studies.

## Materials and methods

### Data availability

The experimental data were mostly one-colour microarray data^12,17,21,22^, with the exception of the *U. minor* datasets^24^, which originated from an RNA-seq experiment (Fig. 1a). All previously published transcriptome raw data are available at the Gene Expression Omnibus (GEO) database under the Accession no. given in Fig. 1a. The full results of the GAGE analysis are provided in the Supplementary Information as Excel files. The egg-induced transcriptome data of *N. attenuata* one day after egg deposition by *S. exigua* is published with this manuscript (NCBI GEO accession GSE148927), along with updated GO term annotations for the *S. dulcamara* and *N. attenuata* transcriptomes (https://primedb.mpimp-golm.mpg.de/index.html?sid=reviewer&pid=2dba617cfed75ebcd6304c236d8b5022).

### Experimental setup

For all plant–insect combinations, we compared the transcriptional response to (a) insect eggs (Fig. 1a, yellow arrows), (b) larval feeding, and (c) eggs and subsequent feeding (Fig. 1a, red arrows). The transcriptomes of leaves exposed to the eggs (E) were compared to control (C) leaves (E vs. C). Transcriptomes from leaves exposed to herbivore feeding (F) and those from leaves exposed to eggs and feeding (EF) were compared to control leaves (F vs. C), and with one another (EF vs. F) (Fig. 1a). Details of the harvesting and plant growth conditions are described in the respective publications. The newly published data on *N. attenuata*’s response to *S. exigua* eggs were generated using the same methodology as described in Drok et al.^21^. These data originate from a leaf systemic to the egg deposition site; the leaf was harvested one day after oviposition. Altogether, we analysed six datasets from plants, which were harvested during exposure to the eggs (C, E). Here, we studied four plant–insect combinations. Furthermore, we analysed seven datasets from plants, which were harvested at a later time when the plants were no longer exposed to eggs (C, F, EF) (Fig. 1a). These latter analyses were carried out for five plant–insect combinations.

We could not include the transcriptome analysis of *Brassica nigra* in response to *P. brassicae* eggs^16^ because this study used two-colour microarrays and therefore does not allow for the direct comparison of feeding-damaged plants with and without prior egg deposition.

### Raw data processing and filtering

As the data originated from different platforms, we normalised and re-processed all raw data. Microarray data were processed with the “limma” package^57^ from Bioconductor^58,59^ in R^60^. Control spots and probes with very low signals were excluded. A signal value of 1.5 times the 90% quantile of the structural dark corner spots on the respective array was set as the detection threshold. Probe signals below this threshold in all samples of at least one of the treatments were removed from the dataset. Data were then background corrected, normalised between arrays using the “normexp” and “quantile” methods, and probes were averaged by their gene identifier. The RNA-seq data from *U. minor* were used pre-processed from NCBI GEO accession GSE77985. Variance in the datasets was visualised by plotting each transcript’s signal standard deviation relative to the mean signal across all treatments (sd/mean). For 20% of the data in all datasets, we found variability in expression that was distinguishable from baseline noise. We chose a conservative approach for variance filtering to reduce the interference from background noise while ensuring that all relevant data would be included in the analysis. Thus, we included in the analysis those 40% of the data that showed the highest variability, although half of these signals still showed minimal variation between treatments. The remaining 60% of the transcripts from all datasets that were most invariant (< 7% variation across the treatments) were excluded. Considering that the classical GO enrichment in the original publications included, at most, 11% of the transcripts measured, we made use of a significantly larger fraction of the data. All processing steps were performed separately for each of the 13 datasets of simultaneously harvested plants (Fig. 1).

### Gene annotation

Genes in the *A. thaliana* gene set were annotated according to TAIR10 using the Bioconductor “GO.db” database^61,62^. Genes in the *U. minor* datasets had recently been annotated based on *A. thaliana* TAIR 10 homologues^24^.

For annotation of the *S. dulcamara*^63^ and *N. attenuata* transcriptomes (BioProject PRJNA223344 at https://www.ncbi.nlm.nih.gov/), we performed BLASTX analyses against protein databases of *Solanum lycopersicum* and *A. thaliana* using NCBI BLAST + software. Protein data were obtained from EnsemblPlants (https://plants.ensembl.org) for *A. thaliana* (TAIR10) and for *S. lycopersicum* (ITAG3.2) from the Sol Genomics Network (https://solgenomics.net/). We considered only the best hits with an E-value ≤ 1e^−5^ as a homologue.

### Gene ontology term annotation

To maximise comparability between species, we assigned GO terms according to TAIR10 homologues via the “GO.db” database^61^. If no suitable TAIR10 match was available for transcripts of the two solanaceous species *S. dulcamara* and *N. attenuata*, we assigned GO terms according to the matching *S. lycopersicum* (ITAG 3.2) homologue.

For the TAIR GO terms assigned via the Bioconductor “GO.db” database^61^, the complete ancestral history of the GO terms was already included. For ITAG3.2 GO terms, however, only the most specific GO terms were provided in the data and all ancestral GO terms were generated using the GOBPANCESTOR function of the “GO.db” package. *Solanum lycopersicum-*specific GO terms, which do not exist in the TAIR database, were removed from the analysis to ensure comparability between datasets (these comprised less than 1% of the assigned GO terms). Depending on the plant species, we were able to assign between 34% (*U. minor*) and 55% (*N. attenuata*) of the processed data to at least one GO term. Those data were used for GAGE analysis.

### Generally applicable gene set enrichment (GAGE)

Gene set enrichment analysis was conducted using the Bioconductor package “gage,” version 2.30^23^. For the analysis, GO terms with at least three, and at most 1,500, genes were defined as gene sets (GS). This approach resulted in 2669 and 2855 GS for the harvest time point during egg exposure and larval feeding, respectively, all of which were included in the analysis of at least one plant–insect combination (Supplementary Table S5). GAGE analyses were conducted separately for up- and down-regulated genes in pairwise treatment comparisons using fold-change in signal as a quantitative parameter and Stouffer’s method^23^ to calculate individual *p*-values from t-statistics, with additional FDR correction. For the *S. dulcamara* datasets, paired t-statistics were used because for each of the three replicates, plants originating from a different European population were used in a full-factorial design. Albeit p-values are corrected for FDR, GAGE analyses can still potentially generate comparatively high false-positive rates^64^. Thus, we suggest using the data provided here to search for conserved patterns across multiple plant–insect interactions and to devise hypotheses for future research. We recommend applying additional methods to verify the differential expression of particular GS that are of interest in only one or few plant–insect combinations.

Since GAGE calculates an enrichment score based on the overall change in expression of genes within each gene set, we refer to GS enriched in transcripts more abundant in egg-laden plants (E vs. C) as *up-regulated* and to those enriched in transcripts less abundant in egg-laden plants as *down-regulated* GS.

### Visualisation and classification of gene set enrichments

For the untargeted comparison of the plant–insect combinations (Figs. 2, 4, 5), we included only those GS that were a part of the GAGE analyses (i.e. a FDR-corrected *p*-value had been assigned to them) in all plant–insect combinations and at all time points of the *U. minor* datasets (1541 GS for the time point during egg exposure and 1548 for the time point during larval feeding, Supplementary Table S6). Of those, we depicted all GS significantly enriched (FDR < 0.05) in at least one plant–insect combination (i.e. in *A. thaliana* with *P. brassicae*, *N. attenuata* with either *M. sexta* or *S. exigua*, *S. dulcamara* with *S. exigua*, or in at least one of the time points in the *U. minor* and *X. luteola* combinations; Fig. 1b, Supplementary Table S3). To facilitate comprehensible visualization of our results, we classified the GS into functional categories that are of general interest to the field. As this classification is partially subjective, the individual functional annotation of all the GS in the conformable response can be found in Supplementary Table S3. As conformably enriched across the plant–insect combinations, we defined GS enriched in at least four of the five combinations (in response to eggs in three out of four, since the *N. attenuata* response to *M. sexta* eggs was not available; Fig. 1b).

FDR-corrected *p*-values were visualised in heatmaps with the R package “ComplexHeatmap”^65^. Venn diagrams were designed with the R-package “eulerr”^66^.

### Additional evaluation of *Solanum dulcamara* transcriptome data

In comparison to the other plant species, the transcriptional response of *S. dulcamara* to herbivore feeding is weaker, and this is especially apparent in the regulation of the phytohormonal pathways (Figs. 2 and 3, F vs. C). In a previous experiment with *S. dulcamara* involving 24 h of feeding by larger (3rd instar) *S. exigua* larvae ^45^, the reported classical GO enrichment matched those of the other plant–insect combinations more closely (Supplementary Fig. S1). To exclude the possibility that the rather weak response of *S. dulcamara* detected here with GAGE in the data from Geuss et al.^22^ was a result of the new annotation or of the data processing methodology, we re-analysed data from Lortzing et al.^45^ using the same filtering and GAGE parameters as described in this paper to the data from Geuss et al.^22^. We re-generated Fig. 3 by replacing the data for F vs. C from Geuss et al.^22^ with those of F vs. C from Lortzing et al.^45^ (Supplementary Fig. S1). This re-analysis resulted in an enrichment pattern which resembles that of the other plant–insect combinations much more closely. Thus, *S. dulcamara’*s weak response to herbivory in the present dataset might result from experimental differences (e.g. less severe feeding damage) between this plant species—herbivore combination and the other plant—insect combinations analysed, but it could also indicate a less severe response of *S. dulcamara* to larval feeding in general. Therefore, the partial lack of otherwise conformable transcriptional responses to feeding between the plant–insect combinations does not necessarily imply a species-specific peculiarity of *S. dulcamara*.

## Supplementary information

Supplementary Information 1.

Supplementary Figure S1.

Supplementary Table S1.

Supplementary Table S2.

Supplementary Table S3.

Supplementary Table S4.

Supplementary Table S5.

Supplementary Table S6.
