# Supplementary material for: Arabidopsis, tobacco, nightshade and elm take insect eggs as herbivore alarm and show similar transcriptomic alarm responses
Source: Sci Rep. 2020 Oct 1;10:16281. doi: 10.1038/s41598-020-72955-y (PMC7530724; doi:10.1038/s41598-020-72955-y)
Supplement: Supplementary file 1 — Supplementary Information 1. [file 41598_2020_72955_MOESM1_ESM.docx]

# Supplementary Information

***Arabidopsis, tobacco, nightshade and*** ***elm take insect eggs as herbivore alarm and show similar transcriptomic alarm responses***

Tobias Lortzing, Reinhard Kunze, Anke Steppuhn, Monika Hilker, Vivien Lortzing

**Tables and Figures**

**Figure S1** Transcriptome data from on *Solanum dulcamara* in response to 24 hours of *Spodoptera exigua* larval feeding (3^rd^ instar) were re-analysed and integrated into the comparative enrichment analysis of gene sets (GS) related to phytohormones. The Fig. S1 is identical to Fig. 3B (F vs C comparison) but the data for *S.dulcamara* (Sd) from Geuss et al. (2018)^1^ are replaced with the data from Lortzing et al. (2017)^2^. The heatmap depicts false discovery rate adjusted *p*-values (FDR) according to the colour key for up- or down-regulated GS. Black colour indicates gene sets which could not be assigned to the plant species. For detailed description of the phytohormone-related gene sets 1H-71H see Supplementary Table S4. Abbreviations: ABA: abscisic acid; AUX: auxin; CK: cytokinin; ET: ethylene; GA: gibberellic acid; JA: jasmonic acid; SA: salicylic acid.

†At: *Arabidopsis thaliana-Pieris brassicae*; Na-M: *Nicotiana attenuata-Manduca sexta* Na-S: *N. attenuata-Spodoptera exigua*; Sd: *Solanum dulcamara-S. exigua*; Um-1h/-6h/-24h: *Ulmus minor-Xanthogaleruca luteola* after 1/6/24 hours of egg deposition and larval feeding, respectively, NA: not annotated. For detailed experimental setup description, see Fig. 1.

**Table S1** Complete case-filtered enrichment analyses of gene sets significantly up- or down-regulated in at least one of the plant-insect interactions† (species are given below) in response to insect eggs (E vs C), larval feeding (F vs C), eggs with subsequent feeding (EF vs C) and the alterations in plant responses to feeding by prior egg deposition (EF vs F). Values indicate FDR-adjusted *p*-values.

†At: *Arabidopsis thaliana-Pieris brassicae*; Na-M: *Nicotiana attenuata-Manduca sexta* Na-S: *N. attenuata-Spodoptera exigua*; Sd: *Solanum dulcamara-S. exigua*; Um-1h/-6h/-24h: *Ulmus minor-Xanthogaleruca luteola* after 1/6/24 hours of egg deposition and larval feeding, respectively. For detailed experimental setup description, see Fig. 1.

**Table S2** Abbreviations for gene sets depicted in Fig. 2 & 5.

**Table S3** Abbreviations for gene sets depicted in Fig. 3 (hormone no.) & 6 (phenylpropanoid no.).

**Table S4** Gene sets (GS) depicted in the Venn diagrams of Fig. 4. GS are up-regulated or down-regulated in different plant-insect interactions† (species are given below) in response to insect eggs (E vs C) or larval feeding (F vs C) (tab E vs C-F vs C) or eggs and larval feeding (EF vs F) (tab E vs C-F vs C-EF vs F).

†At: *Arabidopsis thaliana-Pieris brassicae*; Na-M: *Nicotiana attenuata-Manduca sexta* Na-S: *N. attenuata-Spodoptera exigua*; Sd: *Solanum dulcamara-S. exigua*; Um-1h/-6h/-24h: *Ulmus minor-Xanthogaleruca luteola* after 1/6/24 hours of egg deposition and larval feeding, respectively. For detailed experimental setup description, see Fig. 1.

**Table S5** Overall analyses of gene sets up- or down-regulated in the different plant-insect combinations† (species are given below) in response to insect eggs (E vs C), larval feeding (F vs C), eggs with subsequent feeding (EF vs C) and the alterations in plant responses to feeding by prior egg deposition (EF vs F). Values indicate FDR-adjusted *p*-values. NA: No value was assignable.

†At: *Arabidopsis thaliana-Pieris brassicae*; Na-M: *Nicotiana attenuata-Manduca sexta* Na-S: *N. attenuata-Spodoptera exigua*; Sd: *Solanum dulcamara-S. exigua*; Um-1h/-6h/-24h: *Ulmus minor-Xanthogaleruca luteola* after 1/6/24 hours of egg deposition and larval feeding, respectively. NA: not annotated. For detailed experimental setup description, see Fig. 1.

**Table S6** Complete case-filtered enrichment analyses of up- and down-regulated gene sets in different plant-insect interactions† (species are given below) in response to insect eggs (E vs C), larval feeding (F vs C), eggs with subsequent feeding (EF vs C) and the alterations in plant responses to feeding by prior egg deposition (EF vs F). Values indicate FDR-adjusted *p*-values.

†At: *Arabidopsis thaliana-Pieris brassicae*; Na-M: *Nicotiana attenuata-Manduca sexta* Na-S: *N. attenuata-Spodoptera exigua*; Sd: *Solanum dulcamara-S. exigua*; Um-1h/-6h/-24h: *Ulmus minor-Xanthogaleruca luteola* after 1/6/24 hours of egg deposition and larval feeding, respectively. For detailed experimental setup description, see Fig. 1.

# References

1. Geuss, D., Lortzing, T., Schwachtje, J., Kopka, J. & Steppuhn, A. Oviposition by Spodoptera exigua on Solanum dulcamara Alters the Plant’s Response to Herbivory and Impairs Larval Performance. *Int. J. Mol. Sci.* **19**, 4008 (2018).

2. Lortzing, T. *et al.* Transcriptomic responses of *Solanum dulcamara* to natural and simulated herbivory. *Mol. Ecol. Resour.* (2017). doi:10.1111/1755-0998.12687
