# Supplementary figures and images for: Arabidopsis, tobacco, nightshade and elm take insect eggs as herbivore alarm and show similar transcriptomic alarm responses
Source: Sci Rep. 2020 Oct 1;10:16281. doi: 10.1038/s41598-020-72955-y (PMC7530724; doi:10.1038/s41598-020-72955-y)

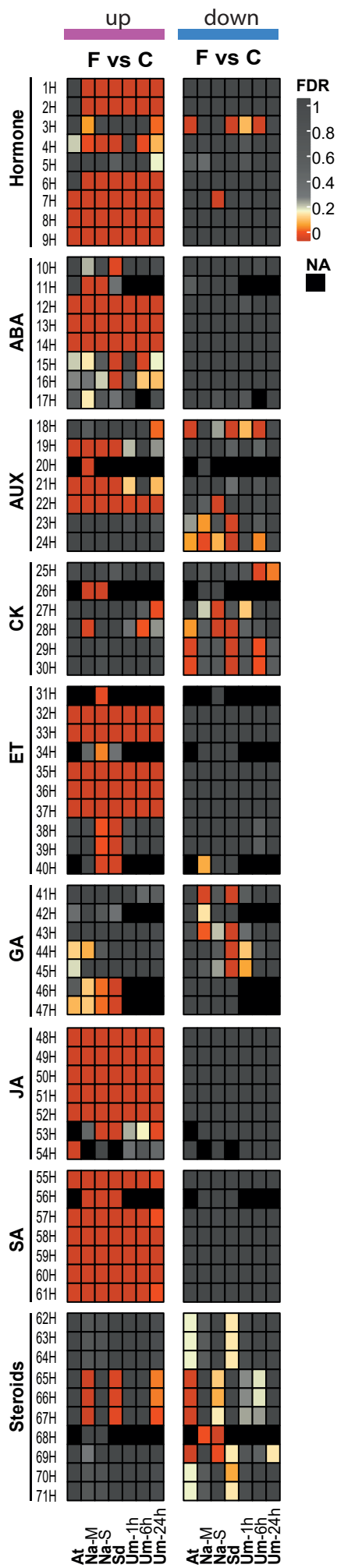

Supplement: Supplementary file 2 — Supplementary Figure S1. [file 41598_2020_72955_MOESM2_ESM.pdf]
